# Autoimmune pulmonary alveolar proteinosis with a history of vaping and vitamin E‐positive bronchoalveolar lavage

**DOI:** 10.1002/rcr2.864

**Published:** 2021-10-18

**Authors:** Tzy Harn Chua, Angela Takano, Yi Ju Yao, Sau Yee Chow, Anantham Devanand, Chee Kiang Tay

**Affiliations:** ^1^ Department of Anatomical Pathology Singapore General Hospital Singapore; ^2^ Duke‐NUS Medical School Singapore; ^3^ Analytical Toxicology Division, Applied Sciences Group Health Sciences Authority Singapore; ^4^ Department of Anesthesiology Singapore General Hospital Singapore; ^5^ Department of Respiratory and Critical Care Medicine Singapore General Hospital Singapore

**Keywords:** autoimmune, pulmonary alveolar proteinosis, vaping, vitamin E, vitamin E acetate

## Abstract

Pulmonary alveolar proteinosis (PAP) can be due to primary autoimmune and secondary causes, including e‐cigarette, or vaping, product use‐associated lung injury. We present a 33‐year‐old male presenting with PAP and a history of vaping. Serum anti‐granulocyte‐macrophage colony‐stimulating factor antibodies were present. Vitamin E (VE), but not VE acetate, was detected in bronchoalveolar lavage. This is the first report of potential association between vaping and autoimmune PAP.

## Graphical abstract

We present a 33‐year‐old male presenting with pulmonary alveolar proteinosis (PAP) and a history of vaping. Serum anti‐granulocyte‐macrophage colony‐stimulating factor antibodies were present. Vitamin E (VE), but not VE acetate, was detected in bronchoalveolar lavage. This is the first report of potential association between vaping and autoimmune PAP.

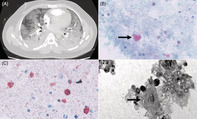

## INTRODUCTION

Pulmonary alveolar proteinosis (PAP) is a rare disease (incidence of 0.24–0.49 per million),^1^ pathologically characterized by alveolar accumulation of surfactant. Autoimmune PAP (APAP), representing 90% of cases, occurs from granulocyte‐macrophage colony‐stimulating factor (GM‐CSF) signalling disruption by serum anti‐GM‐CSF antibodies. Genetic/secondary causes account for the remaining 10%.^1^ Electronic cigarettes (e‐cigarettes), or vaping, product use‐associated lung injury (EVALI) has previously been associated with secondary PAP (SPAP).^2^ We report a patient with APAP, a history of vaping and vitamin E (VE) in bronchoalveolar lavage (BAL) fluid (BALF).

## CASE REPORT

**FIGURE 1:**
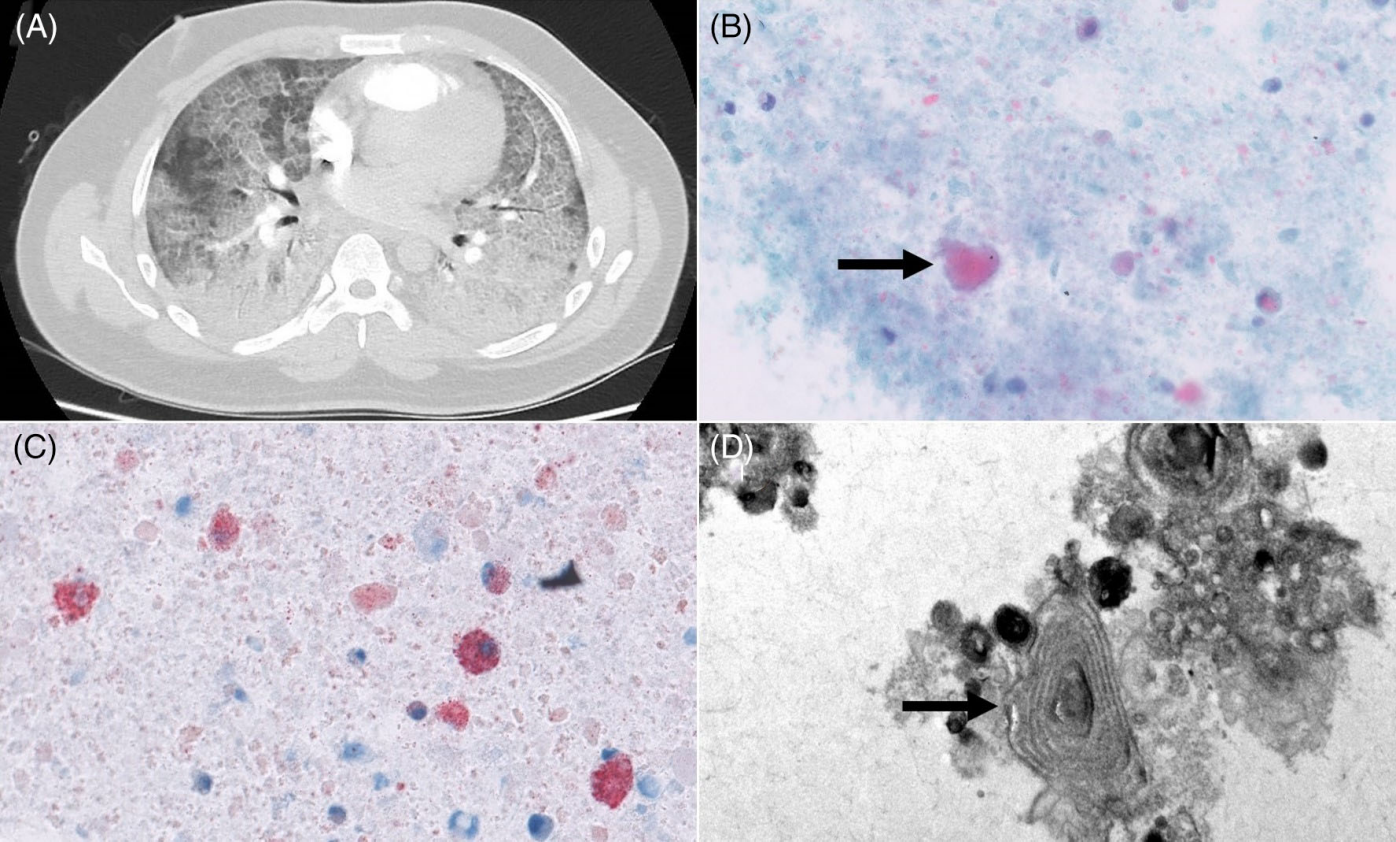
(A) Computed tomography scan showing ground‐glass changes with interlobular septal thickening (crazy‐paving pattern). (B) Amorphous globular structures that stained orange to green (arrow) (Papanicolaou stain, original magnification ×400). (C) Numerous lipid‐laden macrophages and droplets of fat in the background (Oil‐Red‐O stain, original magnification ×400). (D) Lamellar bodies (arrow) seen on electron microscopy (original magnification ×15,000)

A 33‐year‐old male, diagnosed with interstitial lung disease 2 months ago at another institution, presented with worsening dyspnoea from baseline for a week. He was an active smoker (10 pack‐years) and worked as a private hire driver. He had previously used inhalational cannabis and e‐cigarettes, but the frequency and last vaping were unclear. Physical examination revealed bilateral lung crepitations without any signs of underlying connective tissue disease and/or vasculitis. Chest radiography and computed tomography (CT) showed bilateral reticular infiltrates and crazy‐paving pattern (Figure 1A), respectively. Relying solely on the autoimmune work‐up (positive antibodies against signal recognition particle), empiric corticosteroids were started.

On presentation to our hospital, the patient was in type 1 respiratory failure (arterial‐to‐inspired oxygen [PaO_2_:FiO_2_] ratio 90). Despite high‐flow nasal cannula, broad‐spectrum antibiotics and intravenous corticosteroids, his respiratory status deteriorated, requiring endotracheal intubation. BAL recovered opaque and milky BALF. Microbiology (acid‐fast bacilli, fungal organisms, pertinent herpesviruses and respiratory viruses) was unyielding.

BALF cytology showed alveolar macrophages, polymorphonuclear cells and lymphocytes. On Papanicolaou stain, amorphous globular structures staining orange to green (Figure 1B) were discerned. Oil‐Red‐O stain revealed lipid‐laden macrophages (LLM) among fat droplets (Figure 1C), with an LLM index (based on scoring the fat content of 100 consecutive macrophages) of 250. Electron microscopy demonstrated lamellar bodies (Figure 1D) within macrophages and in the background. Gas chromatography‐tandem mass spectrometry (VE acetate [VEA] limit of detection: 1 ng/ml; validated by Health Sciences Authority, Singapore) detected VE but not VEA in BALF. VE concentration was not quantified.

The patient underwent whole lung lavage with dramatic improvements in dyspnoea, oxygen saturation and chest radiography. The serum anti‐GM‐CSF antibodies test was positive via bead‐based multiplex immunoassay at the Mayo Clinic Laboratories in Rochester, with a reference value of less than 15.0 pg/ml deemed to be negative. Genetic testing for hereditary PAP (Invitae Boosted Exome [exome analysis including *ABCA3*, *CSF2RB*, *SFTPB* and *SFTPC* genes]; Invitae panel testing [including *CSF2RA* and *CFTR* genes]) was unremarkable. The final diagnosis was APAP.

## DISCUSSION

This is hitherto the first report of APAP associated with vaping and VE‐positive BALF.

While 50%–80% of APAP affects current or ex‐cigarette smokers,^3^ its association with e‐cigarettes has not been reported. Notably, only one case of SPAP has been linked to vaping e‐cigarettes.^2^ VEA, a condensing chemical commonly found in vaping products, is implicated as a causative agent in EVALI from findings that have emerged from the United States,^4^ where a multi‐centre study involving 51 EVALI patients from 16 states showed that VEA was identified in 94.0% of these patients.^5^ In our patient, the biologically non‐active ester, VEA, could arguably have undergone hydrolysis into its active free form,^6^ explaining his BALF findings. We recognize that BALF VE detection is neither specific for vaping/EVALI.^7^ However, it is plausible/likely that other undiscovered agents may have a role in EVALI's pathogenesis,^8^ considering (1) VEA is absent in vaping products in the United Kingdom and (2) its strong link with EVALI was primarily reported in the United States.^9^

Besides smokers, APAP has been reported in patients with a history of toxic dust exposure/inhalation.^10^ It is postulated that inorganic dust (e.g., indium) may trigger an autoimmune response to cause PAP, although the mechanism remains speculative. Nonetheless, vaping might have influenced the development of APAP in our patient.

PAP typically affects adults aged 30–60 years with a 2:1 male‐to‐female ratio.^1^ Exertional dyspnoea and cough are the most common symptoms. APAP is diagnosed by positive serum anti‐GM‐CSF antibodies, which impair macrophages' ability to clear surfactant. Conversely, autoantibody‐negative SPAP arises from the underlying haematological/autoimmune diseases, infections, malignancy or dust exposure.^1^, ^11^ The ‘crazy‐paving’ appearance on high‐resolution CT is non‐specific despite occurring in a majority (83%) of APAP patients. Radiological differentials include pulmonary oedema/haemorrhage and lipoid pneumonia.^1^ BALF cytology revealing LLM is also diagnostically non‐specific.^1^, ^11^, ^12^

Similarly, EVALI‐related histopathological findings, that is, acute lung injury patterns (e.g., organizing acute lung injury, diffuse alveolar damage and LLM),^13^ are non‐specific; diagnosis relies on the exclusion of infection and alternative causes.

This study describes a patient with APAP with a history of vaping, which had been reported only in SPAP. We postulate that vaping products may act as adjuvants that interact with endogenous proteins to incite self‐reactivity, indirectly triggering the development of APAP.^14^

## CONFLICT OF INTEREST

None declared.

## AUTHOR CONTRIBUTION

Tzy Harn Chua, Angela Takano and Chee Kiang Tay conceptualized the paper and wrote the first draft of manuscript. All authors contributed to the critical revision of the manuscript and approved the final version of manuscript.

## ETHICS STATEMENT

The authors declare that appropriate written informed consent was obtained for the publication of this case report and accompanying images.
